# How to measure person-centred practice – An analysis of reviews of the literature

**DOI:** 10.4102/phcfm.v12i1.2170

**Published:** 2020-03-04

**Authors:** Jakobus M. Louw, Tessa S. Marcus, Johannes F.M. Hugo

**Affiliations:** 1 Department of Family Medicine, School of Medicine, Faculty of Health Sciences, University of Pretoria, Pretoria, South Africa

**Keywords:** review, psychometric properties, measurement instruments, person-centeredness, patient-centeredness

## Abstract

**Background:**

Facilitation and collaboration differentiates person-centred practice (PcP) from biomedical practice. In PcP, a person-centred consultation requires clinicians to juggle three processes: facilitation, clinical reasoning and collaboration. How best to measure PcP in these processes remains a challenge.

**Aim:**

To assess the measurement of facilitation and collaboration in selected reviews of PcP instruments.

**Methods:**

Ovid Medline and Google Scholar were searched for review articles evaluating measurement instruments of patient-centredness or person-centredness in the medical consultation.

**Results:**

Six of the nine review articles were selected for analysis. Those articles considered the psychometric properties and rigour of evaluation of reviewed instruments. Mostly, the articles did not find instruments with good evidence of reliability and validity. Evaluations in South Africa rendered poor psychometric properties. Tools were often not transferable to other socio-cultural-linguistic contexts, both with and without adaptation.

**Conclusion:**

The multiplicity of measurement tools is a product of many dimensions of person-centredness, which can be approached from many perspectives and in many service scenarios inside and outside the medical consultation. Extensive research into the myriad instruments found no single valid and reliable measurement tool that can be recommended for general use. The best hope for developing one is to focus on a specific scenario, conduct a systematic literature review, combine the best items from existing tools, involve multiple disciplines and test the tool in real-life situations.

## Introduction

The applicability, implementation and measurement of person-centred practice (PcP) need to be carefully considered as part of a drive towards universal health coverage, as it brings with it a number of benefits (Table 1), particularly improved patient health outcomes,^1,2,3,4^ as well as a reduction in healthcare provider workload and healthcare service delivery costs.^5,6^ To ensure that these benefits are realised through training, there is a need to accurately measure PcP and that such measurement is based on a well-understood conceptual framework.

**TABLE 1:** Benefits of person-centred practice.

For the patient	For the healthcare system	For the clinician^7^
Higher patient satisfaction^2,7,8^	Better adherence to treatment, recommendations and follow-up visits^1,7,9,10^	More satisfaction
Improved patient health^4,5,7,8,9^	Increased efficiency of care^5^	Better use of time
Improved quality of care^8^	Less hospitalisations^9^	Fewer complaints from patients
More use of preventative care^9^	Shorter hospital stays^4^	-
Better functional performance^4^	-	-
Increased patient engagement^2^	-	-

Person-centredness and patient-centredness are used interchangeably here^11,12^ because of an absence of a universally agreed definition and conceptual similarities described previously.^13^

**FIGURE 1:**
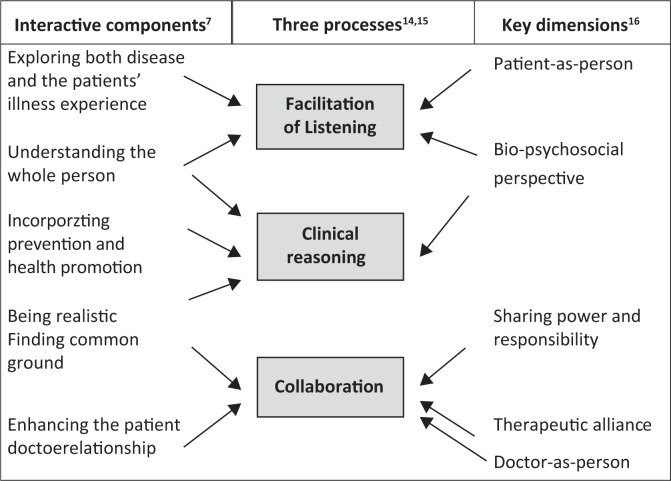
Patient-centred care: Interactive components and key dimensions as related to the three processes of consultation.

‘The clinician as juggler’ used to teach consultation skills at the University of Pretoria^14^ relates well to other frameworks of PcP (Figure 1). The metaphor describes three processes that the clinician has to manage concurrently – facilitation (listening), clinical reasoning (thinking) and collaboration (shared decision-making). The clinician juggling three balls helps us understand the simultaneity and interplay between the three processes.^14,15^

**FIGURE 2:**
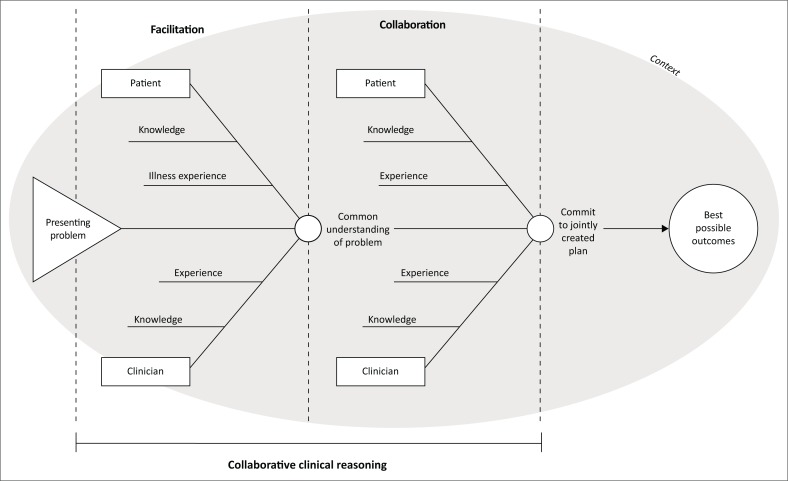
Facilitation, clinical reasoning and collaboration in the consultation.

The clinician must be constantly aware of where each process is, its trajectory and how next to interact with it. The position and trajectory of each process also informs the clinician as to what to do with the others.^14,15^ In this way, he or she brings together clinical expertise and experience with patients’ ideas (Figure 2).^17^

As illustrated in Figure 1, concepts such as ‘patient-as-person’,^16^ ‘exploring the patient’s illness experience’ and ‘understanding the whole person’^7^ manifest themselves in the process of facilitation. Facilitation (caring) is a prerequisite for collaboration. Measuring collaboration may, therefore, indirectly also measure facilitation.

The process of collaboration in the consultation is related to the concepts of ‘sharing power and responsibility’, ‘therapeutic alliance’,^16^ ‘finding common ground’ and, to some extent, ‘enhancing the patient-doctor relationship’^7^ (Figure 1). Collaboration can be measured by the degree to which the clinician explains the risks and side effects of management options, explores the patient’s questions and expectations, and plans with the patient so that he or she understands and is willing and able to follow it. Because competency in clinical reasoning is the foundation of collaboration with a patient, collaboration can serve as an indirect measure of clinical reasoning. Collaboration is thus an outcome of PcP.^18^

The discovery of a patient’s perspective and shared control of the consultation are in fact the two features that distinguish a person-centred consultation from a traditional biomedical consultation.^19^ Research suggests that it is patients’ perceptions of PcP that correlate best with improved health outcomes associated with PcP.^3,5,9,20^ This is because an adequate biopsychosocial understanding enables the clinician and the patient to consider relevant and possible management options within the patient’s specific context and preference, thereby saving valuable time in the consultation, ensuring patient-relevant solutions and better contributing to health and treatment outcomes.

Measuring person-centredness is difficult,^21,22^ evidenced by the sheer volume of measurement tools developed, published and evaluated in various contexts. Many of these measure subcomponents of person-centred care, while several attempt to measure the concept as a whole. Some are specifically designed to evaluate a single visit to a healthcare practitioner, while others try to measure person-centredness over a period of time.^22^

While numerous reviews of instruments have been performed, the aim of this article was to assess the measurement of facilitation and collaboration in selected reviews of PcP instruments, as these are elemental components in all frameworks of person-centred consultations.^13^

## Methods

Literature searches were conducted from 01 January 2000 to 02 May 2019 in Ovid Medline and Google Scholar. Search terms used include patient-centredness, patient-centred, person-centredness, person-centred combined with measurement tools or instruments, evaluate or evaluation, and assessment. The search yielded 13 548 articles in Ovid Medline, 83 of which were English language review articles with structured abstracts applicable to adults. References in and citations of relevant articles were screened to identify additional review articles. The first author screened review articles by their titles. Inclusion criteria were comparison of instruments that measure person- or patient-centredness in the medical consultation. Exclusion criteria were being in a language other than English, not being review articles, not comparing measurement instruments, no structured abstract, not referring to adult patients and an exclusive focus on a specific disease (e.g., epilepsy) or discipline such as gerontology, oncology and palliative care.

Eligible review articles were then thematically analysed by the first author to specifically consider the measurement of facilitation and collaboration in the medial consultation, as well as the psychometric properties of the instruments reviewed. Measurement items in preferred tools identified in the review articles were classified by the first author as related to collaboration, facilitation or clinical reasoning. For the items from the first tool so analysed two experienced family physicians (the third author and another) reviewed this classification of measurement items. Differences were discussed until consensus was reached.

### Ethical considerations

This review is part of a PhD thesis entitled “Learning of person-centred practice amongst clinical associate students at the University of Pretoria”. It was approved by the Research Ethics Committee of the Faculty of Health Sciences, University of Pretoria, reference number 128/2013.

## Results

**FIGURE 3:**
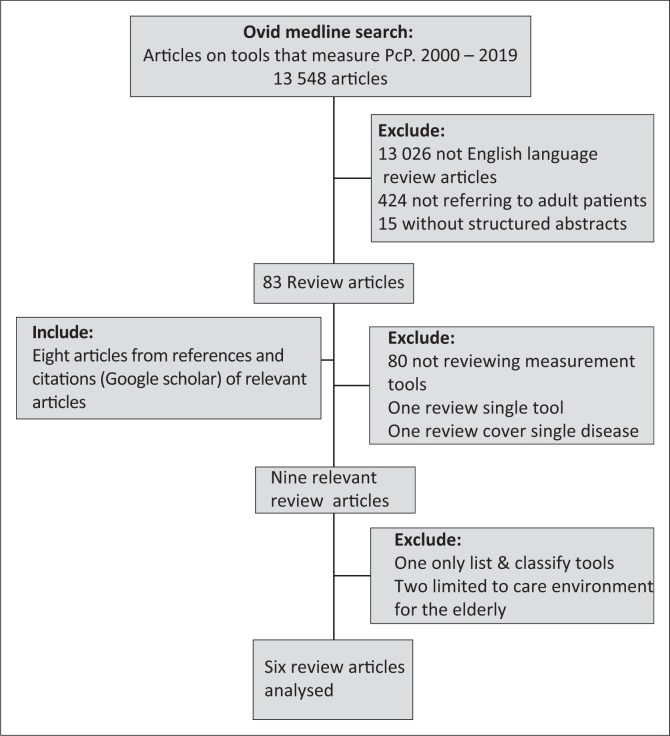
Search and selection of articles.

Nine review articles published in the period 2010–2018 were identified (Figure 3). One of these was a rapid review, listing and classifying 160 tools to measure person-centred care without evaluating their quality.^23^ In the remaining eight articles, 129 measurement tools were reviewed. Two of the tools appeared in three reviews and 11 in two reviews, while the remaining 116 were only included once in a review. The analyses by Edvardsson et al.^24^ and Wilberforce et al.^25^ were subsequently also excluded as they reviewed tools that measure the person-centredness of the care environment of people with dementia and older people, but not of medical consultations.

This analysis is based on the remaining six review articles^20,22,26,27,28,29^ in which measurement instruments of PcP in the medical consultation were included. The number of tools reviewed per article varied from 12 to 40. The six reviews are summarised in Table 2 and discussed below.

**TABLE 2:** Summary of six review articles.

Author(s)	Focus	Number of tools reviewed	Assessment of methodological quality and psychometric properties	Preferred measurement tools and reasons preferred				Findings	Recommendations
				Tools	Facil†	Coll‡	*α*§		
Hudon et al.^20^	Self-administered instruments measuring patients’ perceptions of patient-centred care	13	STARD	Measure patient-centredness as concept and scored 11/15 on STARD				The patient needs to perceive that his or her individual needs and circumstances are at the heart of the clinical care he or she receives	Study the convergent validity of patient-centred care instruments (CCM and PPPC) and subscales or items of other instruments
				CCM	11	8	-¶	Higher levels of patient-centred care on CCM and PPPC were associated with better health outcomes in the short term	
				PPPC-14	6	8	0.71^20^		
Zill et al.^26^	Physician–patient communication	20	COSMIN checklist and quality criteria of Terwee et al.	≥2 good or excellent COSMIN ratings and ≥2 positive on Terwee				Most scores on COSMIN fair to poor	Further psychometric evaluation of tools with the COSMIN checklist
				SEGUE	13	13	0.57^31^		
				PBCI	15	2	-¶		
				QQPPI	7	6	0.95^32^		
Brouwers et al.^22^	Instruments for measurement of and feedback on patient-centredness	14	COSMIN checklist	PCOF: Covers all dimensions				The complexity of patient-centredness may hamper measurement and assessment. Situational flexibility and context sensitivity not considered. Most instruments not thoroughly investigated	Further research and enhancement of validity, reliability, generalisability, responsiveness, interpretability in different contexts, comprehensibility and feasibility
				PFC: Excellent validity but reliability not studied					
				CARE: Only one to consider flexibility – according to preference of the patient					
				PCOF	22	30	0.67^33^		
				PFC	6	7	0.89^34^		
				CARE	7	2	0.93^22^		
Al-Jabr et al.^28^	Patient feedback questionnaires that assess the development of consultation skills	12	NIH Quality Assessment Tool	DISQ: Only one valid and reliable on >two criteria				Most studies had poor to fair methodological quality. Feasible to use patient feedback, but the impact on consultation skills development not clear	To use patient feedback to improve consultation skills: Use a valid and reliable questionnaire (e.g. DISQ with at least 25 patients per practitioner) An independent person recruits patients face-to- face Collect patient feedback immediately after the encounter and over more than 1 day Report feedback results to practitioners comparing with peers Follow with reassessment of practitioners
				DISQ	5	1	0.96^35^		
Gärtner et al.^27^	SDM measurement instruments	40	COSMIN checklist, quality criteria of Terwee et al. and best-evidence synthesis	Had positive evidence of at least one type of reliability and one type of validity				Lack of evidence on measurement qualities Positive results where content and structural validity were evaluated but negative results where inter-rater reliability and hypothesis testing were evaluated	Define SDM clearly Determine content validity prior to further validation Large enough sample sizes Improve test–retest and inter-rater reliability Determine minimal important change values Evaluate and refine existing instruments. Adhere to the COSMIN guidelines
				FPI	2	7	0.91^36^		
				SDM-Q-9	0	9	0.94^37^		
Sustersic et al.^29^	Creating a measurement tool from the literature for doctor–patient communication in the emergency department	22	None	High internal consistency and good external validity				Developed a measurement tool: specific scenario, good literature review, good theoretical model, combine items from existing tools, involve multiple disciplines	Test DPC-13 in other clinical situations and populations
				DPC-13	7	5	0.89^29^		

*Source:* Van Tulder M, Furlan A, Bombardier C, Bouter L, Editorial Board of the Cochrane Collaboration Back Review Group. Updated method guidelines for systematic reviews in the cochrane collaboration back review group. Spine (Phila Pa 1976). 2003;28(12):1290–1299. https://doi.org/10.1097/01.BRS.0000065484.95996.AF; Schellingerhout JM, Verhagen AP, Heymans MW, Koes BW, de Vet HC, Terwee CB. Measurement properties of disease-specific questionnaires in patients with neck pain: A systematic review. Qual Life Res. 2012;21(4):659–670. https://doi.org/10.1007/s11136-011-9965-9

CARE, Consultation and Relational Empathy; CCM, Consultation Care Measure; COSMIN, COnsensus-based Standards for the selection of health Measurement INstruments; DISQ, Doctor Interpersonal Skills Questionnaire; DPC, Doctor–Patient Communication; FPI, Facilitation of Patient Involvement in Care; NIH, National Institutes of Health; PBCI, Patient-Centred Behaviour Coding Instrument; PCOF, Patient-Centred Observation Form; PFC, Patient Feedback Questionnaire on Communication Skills; PPPC, Patient Perception of Patient-Centredness; QQPPI, Questionnaire on Quality of Physician–Patient Interaction; SDM, shared decision-making; SEGUE, Set the stage, Elicit information, Give information, Understand the patient’s perspective, and End the encounter; STARD, Standards for Reporting of Diagnostic Accuracy.

† , Number of items in the tool measuring facilitation.

‡ , Number of items in the tool measuring collaboration.

§ , Cronbach’s alpha.

¶ , For two tools, Cronbach’s alpha was reported for subscales only.

Three^22,26,27^ of the six reviews used the COnsensus-based Standards for the selection of health Measurement INstruments (COSMIN) checklist^30^ to evaluate the methodological quality of each study reviewed, while one^20^ used a modified version of the Standards for Reporting of Diagnostic Accuracy (STARD) scale and another^28^ used the National Institutes of Health (NIH) Quality Assessment Tool.

The standard of assessment in evaluating studies of measurement instruments is clearly higher in the later reviews than in the earlier ones. Not only do authors compare the psychometric properties of the various instruments, but they also consider the methodological rigour of the studies that measured those properties. Gärtner et al.^27^ used an adapted scale from the Cochrane Back Group to synthesise both aspects into one rating (Table 3).^38,39^ This made it possible to rate each measurement property (e.g. internal consistency, reliability, measurement error, content validity and structural validity) of each measurement instrument.

**TABLE 3:** Quality synthesis.

Level	Rating	Description
Strong	+++ (−−−)	Consistent positive (negative) ratings derived from multiple studies of good quality, or in one study of excellent quality
Moderate	++ (−−)	Consistent positive (negative) ratings in multiple studies of fair quality, or in one study of good quality
Limited	+ (−)	Positive (negative) rating in one study of fair quality
Conflicting	+/−	Conflicting results
Unknown	?	Only studies of poor quality

*Source:* Van Tulder M, Furlan A, Bombardier C, Bouter L, Editorial Board of the Cochrane Collaboration Back Review Group. Updated method guidelines for systematic reviews in the cochrane collaboration back review group. Spine (Phila Pa 1976). 2003;28(12):1290–1299. https://doi.org/10.1097/01.BRS.0000065484.95996.AF; Schellingerhout JM, Verhagen AP, Heymans MW, Koes BW, de Vet HC, Terwee CB. Measurement properties of disease-specific questionnaires in patients with neck pain: A systematic review. Qual Life Res. 2012;21(4):659–670. https://doi.org/10.1007/s11136-011-9965-9

Gärtner et al.^27^ ascribe the lack of good evidence on the measurement qualities of instruments both to a failure to study their measurement properties and to the poor methodological quality of validation studies. They argue that this does not mean that existing instruments are necessarily of poor quality, only that their quality is often unknown.^27^ Many measurement instruments fail to define the concept that is being measured clearly, and this affects the comparability of results.^27,40^

Most tools have been developed in first-world countries. Of the few tested in Africa, the Physician–Patient Communication Behaviours scale was developed by adapting 19 statements from a matched-pair instrument for local use in Kenya. Patients at anti-retroviral treatment clinics responded to these on a Likert scale. Thirteen statements were found to be reliable and useful in that setting. Another, the Measure of Processes of Care (MPOC) developed in Canada, was tested in seven countries including South Africa. It measures family-centred care provided to children with chronic conditions over the past year by asking parents or caregivers to respond to questions on a Likert scale. After adaptation for resource-poor settings in South Africa (MPOC–22 [SA]),^41^ it was found to be neither reliable nor valid. Of the 22 items tested, the eight that reached an acceptable degree of reliability and validity formed the basis for MPOC–8 (SA), which needs to be studied further. The validity and reliability of the Patient–Practitioner Orientation Scale was found to be poor when evaluated with South African medical students.^40^

Both Zill et al.^26^ and Brouwers et al.^22^ reviewed the Questionnaire on Quality of Physician–Patient Interaction (QQPPI).^32^ They concurred that the internal consistency and construct validity methodology was good, while that for reliability was poor. However, there was some divergence in their assessment of the methodology for measuring content validity. Zill et al.^26^ rated it as poor and Brouwers et al.^22^ as fair.

The Patient Feedback Questionnaire on Communication Skills (PFC)^34^ received three positive ratings with excellent methodological scores for validity.^18^ Reliability has not been tested. However, a study evaluating the PFC^28^ was itself rated on the NIH Quality Assessment Tool for Observational Cohort and Cross Sectional Studies, as ‘poor’ (3/14) with a high risk of bias.

Gärtner et al.^27^ found that only seven of 40 measurement instruments had moderate to strong evidence of positive performance on at least one aspect of each of validity and reliability. Of these, only the Facilitation of Patient Involvement in Care (FPI) is in English and only three (non-English) instruments had no negative scores on other measurement properties.

The Doctor–Patient Communication (DPC) scale of Sustersic et al.^29^ for acute conditions has 13 items with good internal consistency. It is an adaptation of items from 22 measurement tools identified by them in a systematic review and elaborated through a multidisciplinary informed theoretical model.

Many of the tools use similar items to measure PcP. Broadly, they can be grouped into those that relate to facilitation, clinical reasoning and collaboration.

As Table 2 shows, the internal consistency of the better-performing tools is greater when they focus mostly on either facilitation or collaboration. Thus, the four with more than 75% of their items measuring either facilitation or collaboration reported Cronbach’s alpha values above 0.9. Of the six tools with a greater balance of facilitation and collaboration measures, three had Cronbach’s alpha values below 0.75. This finding may be an indication that facilitation and collaboration are not directly correlated. In other words, an increase in one may not be accompanied by an increase in the other. Or, equally, that some clinicians may practise one construct more while others practise the other more. Measurement tools that try to measure both may therefore suffer from poor internal consistency.

## Implications and recommendations

In the six reviews of instruments to measure PcP as a whole or its components, only one commits to a single measurement tool (Doctor Interpersonal Skills Questionnaire [DISQ]) as having better evidence of being valid and reliable than others.^28^

On the basis of her rapid review of instruments available to measure PcP, de Silva^23^ concludes that there is no agreement on a single best measure that covers all aspects of person-centred care. Instead, she recommends combining and testing various measurement methods and tools locally to determine their local usefulness.

Reviews call for more studies with adequate methodological rigour to evaluate the psychometric properties of measurement instruments. Three^22,26,27^ that used the COSMIN checklist recommend its use while one^22^ found it to be in need of further development and testing.

Rather than developing new instruments, the reviews recommend that researchers focus on refining existing measurement instruments to improve their validity, reliability, generalisability, responsiveness, comprehensibility and feasibility. In this, attention needs to be paid to aspects of interpretability in different contexts^22,25^ by different practitioners.^28^ Given the association between better health outcomes and patients’ perceptions of patient-centredness,^3,5,9,20^ instrument development also requires inputs from patients and their families.^24,25^ Also, even with excellent translation methods, measurement instruments need to be adapted for and tested in new socio-cultural environments before they are used.^40,41^

In general, instruments should measure the quality of both facilitation and collaboration in the medical consultation, even where combining the two may reduce internal consistency. Furthermore, there is a need to study the reliability and validity of subscales in the instruments, not only of the overall instrument.

In choosing among the 12 tools (Table 2), PcP researchers need to take account of what they seek to measure (facilitation, collaboration or both), who will rate the PcP, and the context, language and population, etc. More than 75% of items in the DISQ, Patient-Centred Behaviour Coding Instrument (PBCI) and Consultation and Relational Empathy (CARE) measure relates to facilitation while more than 75% of items in the nine-item Shared Decision-Making tool (SDM-Q-9) and FPI relate to collaboration. Only the Patient-Centred Observation Form (PCOF), Set the stage, Elicit information, Give information, Understand the patient’s perspective, and End the encounter (SEGUE) and PBCI are designed to be completed by observers, and the rest by the patient. Most tools are only available and validated in English. Some have been translated into other languages but often lost reliability in the process.

Further research into the measurement properties of existing instruments to measure PcP should be guided by the COSMIN checklist. Reviewers of such research should preferably report both the measurement properties and the strength of the evidence for them in a single, well-defined scale.

Should new instruments be needed for specific scenarios or socio-cultural-linguistic contexts, the concept to be measured should firstly be clearly defined before well-performing items from existing instruments can be selected with input from patients, families and experts from various disciplines. For developing a valid and reliable measurement tool, the methodology of Sustersic et al.^29^ can be considered. They focussed on a specific scenario, conducted a thorough systematic literature review of existing applicable tools, combined the best items from such tools, involved multiple disciplines to select and adapt items and tested their new tool in real-life situations.

## Limitations

Because our initial search strategy was limited to two databases, it is possible that some applicable reviews were not identified for this article. However, screening references in and citations of review articles did identify several appropriate reviews.

The first author classified the various items of the measurement tools as pertaining to clinical reasoning, facilitation or collaboration. Only for one tool (SEGUE) was this classification verified by two other experts.

A limitation identified in the tools reviewed was that the voice of patients themselves is usually not included in the development of PcP measurement tools. It seems logical that the best person to measure person-centredness of any healthcare service would be the patient – the one for whom the service exists – because the patient is the one experiencing the person-centredness (or not) of the service and because greater perceptions of person-centredness have a stronger association with improved patient outcomes.^3,5,9,20^ However, account also has to be taken of the fact that patients often rate the service (or actually the providers) highly, in part because they are dependent on the service and may feel vulnerable (fear retribution) and in part because of social desirability, as they just want to be nice and avoid making uncomfortable but true assessments. This limitation notwithstanding, the fact that patients are rarely involved in the development of measurement instruments is a serious ommission.^25^

## Conclusion

The multiplicity of measurement tools is a product of many dimensions of person-centredness that can be measured from many perspectives (patients, family, clinicians and observers) and in many service scenarios inside and outside the medical consultation. In addition, tools are often not transferable to other socio-cultural-linguistic contexts, both with and without adaptation.

In spite of extensive research, there is no single valid and reliable measurement tool that can be recommended for general use. Instruments focussed on patients’ perceptions of PcP may be more useful in outcomes research,^3,5,9,20^ whereas instruments completed by peers or facilitators of learning may be more useful in teaching.^42^

Many tools are developed – often by the same authors – but few are studied extensively in terms of their psychometric properties and usefulness for research on and teaching of person-centredness. Often, a tool is developed, evaluated and then abandoned. This leaves us without measurement tools for which we have good evidence – repeated in several studies – of all their properties. Some are valid, others are reliable, while others are neither. Many are untested.

Using the COSMIN checklist can increase the quality of research even though researchers may sometimes differ in their application of the standard.
